# Genome-wide meta-analyses of cross substance use disorders in European, African, and Latino ancestry populations

**DOI:** 10.21203/rs.3.rs-3955955/v1

**Published:** 2024-07-16

**Authors:** Dongbing Lai, Michael Zhang, Nick Green, Marco Abreu, Tae-Hwi Schwantes-An, Clarissa Parker, Shanshan Zhang, Fulai Jin, Anna Sun, Pengyue Zhang, Howard Edenberg, Yunlong Liu, Tatiana Foroud

**Affiliations:** Department of Medical and Molecular Genetics, Indiana University School of Medicine; Indiana University School of Medicine; Indiana University School of Medicine; Indiana University; Department of Medical and Molecular Genetics, Indiana University School of Medicine; Middlebury College; Case Western Reserve University; Case Western Reserve University; Indiana University School of Medicine; Indiana University; Indiana University School of Medicine; Indiana University School of Medicine

## Abstract

Genetic risks for substance use disorders (SUDs) are due to both SUD-specific and SUD-shared genes. We performed the largest multivariate analyses to date to search for SUD-shared genes using samples of European (EA), African (AA), and Latino (LA) ancestries. By focusing on variants having cross-SUD and cross-ancestry concordant effects, we identified 45 loci. Through gene-based analyses, gene mapping, and gene prioritization, we identified 250 SUD-shared genes. These genes are highly expressed in amygdala, cortex, hippocampus, hypothalamus, and thalamus, primarily in neuronal cells. Cross-SUD concordant variants explained ~ 50% of the heritability of each SUD in EA. The top 5% individuals having the highest polygenic scores were approximately twice as likely to have SUDs as others in EA and LA. Polygenic scores had higher predictability in females than in males in EA. Using real-world data, we identified five drugs targeting identified SUD-shared genes that may be repurposed to treat SUDs.

## Introduction

Substance use disorders (SUDs, including alcohol, cannabis, opioid, and nicotine) have devasting consequences on individuals, their families, and the society. Globally, approximately 5.5% of disability-adjusted life-years are attributable to SUDs^1^. For each SUD, twin and family studies have shown that genetic factors are responsible for ~ 50% of the variation^2^. Searching for SUD-associated genes will not only help us understand the genetic etiologies of SUDs, but it can also facilitate the development of novel prevention and treatment strategies by identifying drugs targeting genes related to SUDs.

SUDs share common features such as uncontrolled use of substances, withdrawal/negative affect, and compromised executive functioning^3^. Many people use more than one substance and suffer from multiple SUDs simultaneously^4^. This comorbidity among SUDs suggested shared genetic components across different SUDs, as demonstrated by twin studies^5–7^ and genetic correlation studies^8,9^. Furthermore, recent large-scale genome-wide association studies (GWAS) of individual SUD have identified genes associated with more than one SUD, e.g., *DRD2*^10,11^, *FTO*^10,12–14^, and *PDE4B*^10,11,13^. To identify SUD-shared genes, one way is to perform univariate analysis by defining SUDs cases as having any SUD and defining controls as not having any SUD. However, individual level genotype data are typically required and thus studies using these methods have small to moderate samples sizes^15–19^, and as a result, their findings remain to be validated. Multivariate methods such as meta-analysis and genomic structural equation modeling (genomic SEM)^20^ can utilize summary statistics from large-scale GWAS and thereby are more powerful. Recently, large-scale studies using genomic SEM to search for SUD-shared genes reported novel associations^8,21^. However, they only explained small portion of genetic variations of SUDs and many SUD-shared genes remain to be discovered^8,21^.

To identify more SUD-shared genes, we performed the largest cross-SUD meta-analyses to date. GWAS summary statistics of problematic alcohol use (PAU)^10,12,22^, cannabis use disorder (CUD)^23^, opioid use disorder (OUD)^24,25^, and nicotine use disorder (NUD)^26,27^ were included. We first performed cross-SUD meta-analyses in European (EA), African (AA), and Latino ancestry (LA) samples. Since many individuals have multiple SUDs simultaneously, it is unlikely that a variant increases the risk for one SUD but decreases the risk for another SUD in the same person. Therefore, we only retained variants that had the same directions of effects in different SUDs (i.e., SUD-concordant variants). We also performed three cross-ancestry meta-analyses in EA and AA (EA_AA), EA and LA (EA_LA), EA, AA, and LA (EA_AA_LA) samples. If a variant is associated with SUDs in different populations, then it likely has the same direction of effect across different populations; therefore, we only retained population-concordant variants in cross-ancestry meta-analyses. These SUDs are correlated and thus we used the fixed-effects meta-analysis instead of genomic SEM as it is more powerful^28–30^. We performed gene-based analyses using SUD-concordant variants, limited to those genes that are expressed in at least one of the 13 brain regions in GTEx^31^. We used multiple approaches to map variants to genes and prioritize mapped genes. Then for prioritized genes, we performed brain dissection and cell-type enrichment analyses using the single-cell RNA expression data from the BRAIN Initiative Cell Census Network (BICCN)^32^. Heritability estimates and genetic correlation analyses were also performed. We generated polygenic scores and tested their predictabilities for SUDs using data from the All of Us research program and the Indiana Biobank^33^. Lastly, we identified drugs targeting prioritized genes then tested whether these drugs could be repurposed to treat SUDs by using real-world data.

## Results

### Sample description.

GWAS summary statistics included in this study are summarized in Table 1. EA GWAS included PAU^10^, CUD^23^, OUD^24,25^, NUD^26,27^ and substance abuse from the FinnGen consortium^34^. Diagnosis of each SUD in these GWAS was based on DSM-IV, ICD 9/10 codes, problematic subscale of the Alcohol Use Disorder Identification Test, and the Fagerstrom Test for Nicotine Dependence. SUDs cases in FinnGen were defined as having any SUD, and controls were those without any SUDs and mental disorders. Sample sizes in EA GWAS ranged from 251,534 to 435,563. AA GWAS included alcohol use disorder (AUD)^12,22^, CUD^23^, OUD^24,25^, and NUD^27^. Sample sizes ranged from 9,745 to 91,026. LA GWAS included AUD (N = 14,175)^12^ and OUD (N = 34,861)^24^. For each SUD, if there was more than one GWAS (e.g., EA OUD GWAS), then they were meta-analyzed first before performing cross-SUD meta-analyses. Sample overlapping across different studies was corrected during meta-analysis. The effective sample sizes after correcting for overlapping samples were 1,467,929 (EA), 159,000 (AA), 45,727 (LA), and 1,672,656 (cross-ancestry total). Using EA samples, the genetic correlations among PAU, OUD, CUD and SUD ranged from 0.48 to 0.70 (P-values ≤ 5.19E-12, **Supplemental Table 1**), indicating shared genetic underpinning across SUDs.

### Identification of loci associated with SUDs.

We defined independent lead variants as those genome-wide significant (GWS) variants with the smallest P-values and linkage disequilibrium (LD) r^2^ < 0.1 with other GWS variants. We defined a significant locus as a chromosome region surrounding an independent lead variant bordered by variants having LD r^2^ > 0.6 with the independent lead variant. If the distance between two loci was < 250 kb, then they were merged. In cross-SUD meta-analyses, the numbers of independent lead variants identified were: 68 (40 loci) in EA, 1 (1 locus) in AA, and 4 (3 loci) in LA (**Supplemental Tables 2–4**). However, the locus in AA was an intergenic variant without any LD support; therefore, it was likely a false positive and was excluded from further analysis. rs1229984 in *ADH1B*, which is a well-known alcohol metabolism gene that is associated with AUD^35^, was identified in LA. LA had only AUD and OUD GWAS and it is likely that this finding was driven by AUD, but we included this locus in subsequent analyses as we cannot rule out its role in other SUDs. In cross-ancestry meta-analysis, the numbers of independent lead variants identified were: 29 (20 loci) in AA_EA, 35 (24 loci) in EA_LA, and 17 (12 loci) in AA_EA_LA (**Supplemental Tables 5–7**). Manhattan plots of cross-SUD and cross-ancestry meta-analyses are in **Supplemental Fig. 1**. By merging loci identified in all meta-analyses that have inter-loci distances < 250 kb, we identified 45 loci in total (Table 2). LocusZoom plots of these 45 loci in different meta-analyses are in **Supplemental Fig. 2**. Among them, 29 loci were identified by multiple meta-analyses (Table 2); and many of them have different independent lead variants in different meta-analyses (**Supplemental Fig. 2**).

There are 104 genes that have at least one GWS variant within its boundaries (transcription start and end sites ± 1 kb, **Supplemental Tables 2–7**). Seven variants change protein products (missense or stop gain): rs11604671 in *ANKK1*, rs11601425 in *TMPRSS5*, rs601338 in *FUT2*, rs2287922 in *RASIP1*, rs1229984 in *ADH1B*, rs3736781 in *BTN1A1*, and rs1057868 in *POR*. *ANKK1* and *TMPRSS5* are ~ 8 kb and ~ 212 kb from *DRD2*, a well-known gene associated with SUDs. *ANKK1* is a part of signal transduction pathway and *TMPRSS5* is a member of the serine protease family. It is noteworthy that the missense variant rs11604671 in *ANKK1* exhibited P-values < 0.05 in all SUD GWAS in EA. *FUT2* and *RASIP1* are ~ 50 kb and ~ 25 kb from *FGF21*, which is related to taste liking measurement and alcohol consumption. *FUT2* encodes the alactoside 2-L-fucosyltransferase enzyme and *RASIP1* is related to GTPase binding and protein homodimerization activities. *BTN1A1* belongs to immunoglobulin superfamily. *POR* is reported as related to coffee and tea consumption in the GWAS catalog^36^. Additionally, there are 10 protein changing variants in nine genes that have LD r^2^ > 0.6 with independent lead variants (**Supplemental Table 8;** all had P-values ≤ 1.85E-06). Seven of them are in *ANKK1, BTN1A1, FUT2, POR, RASIP1,* and *TMPRSS5* but the other three are in different genes (rs61785974 in *PHACTR4*, rs6720 in *MDH2*, rs589292 in *SCAI*). All these genes are reported in the GWAS catalog as associated with SUD-related or neuropsychiatric-related traits^36^.

We only required variants having concordant effects by design; however, some GWS variants also showed certain degree of associations (e.g., P-value < 0.05) across all SUDs. In EA, there were 302 GWS variants with P-value < 0.05 in all five EA SUD GWAS (**Supplemental Table 2**). Among these, 169 are located in 25 genes, with most of them (*ANKK1, ARIH2, DRD2, IHO1, KDM4B, LINC01360, NCAM1, PDE4B, PDE4B-AS1, PPP1R13B-DT, PRKAR2A, QRICH1, RABEPK, RUNX1T1, SLC25A20,* and *USP19*) reported as SUD-associated in the GWAS catalog^36^. No GWS variants had P-value < 0.05 in all cross-ancestry GWAS.

### Gene-based analysis.

MAGMA^37^ was used to perform gene-based analysis within each ancestral population, and gene-based results from each ancestry population were meta-analyzed also using MAGMA^37^. After Bonferroni correction, the numbers of significant genes identified were: 124 in EA, 1 in AA, 2 in LA, 137 in AA_EA, 109 in EA_LA, and 137 in AA_EA_LA (**Supplement tables 9–14**). In total, 169 unique genes were identified by gene-based analysis. Manhattan plots of gene-based analyses are in **Supplemental Fig. 3.**

### Mapping significant variants to genes.

For all GWS variants, we used positional mapping (whether a GWS variant is in a gene), eQTL mapping (whether a GWS variant is a cis-eQTL of a gene) and chromatin interaction mapping (whether a GWS variant interacts with a gene) to identify genes impacted by each variant. Similar to gene-based analysis, we limited to genes that are expressed in at least one brain tissue from GTEx^31^. The numbers of genes identified were: 217 in EA, 0 in AA, 6 in LA, 127 in AA_EA, 148 in EA_LA, and 85 in AA_EA_LA (**Supplemental Tables 15–19**). In total, 244 unique genes were identified.

### Gene prioritization.

The combination of gene-based analysis and gene mapping identified a total of 299 genes. However, some of them may be simply close to SUD-associated genes but not SUD related; therefore, we used two strategies to prioritize mapped genes. First, we checked which genes were in any of nine SUD-related pathways (alcoholism, amphetamine addiction, cocaine addiction, morphine addiction, nicotine addiction, dopaminergic synapse, GABAergic synapse, glutamatergic synapse, and MAPK signaling) from the Kyoto Encyclopedia of Genes and Genomics (KEGG: https://www.genome.jp/kegg/); and nine genes are within these pathways. We also prioritized genes in the same gene families as those in SUDs pathways (12 genes), or that directly interact with genes in SUDs pathways (73 genes) using the STRING database^38^. In total, this strategy prioritized 94 genes (**Supplemental Table 20**). Second, we identified 240 genes in the GWAS catalog^36^ that are associated with any psychiatric trait, brain measurement, and brain function. Together, these two strategies prioritized 250 genes, which include 220 protein coding genes, 15 non-coding RNA, 10 antisense genes, and 5 pseudogenes, (**Supplemental Table 20**). Some genes are worth noting. For instance, *OPRD1*, which was identified by positional mapping, is a member of opioid receptor signaling pathway and was nominated as SUD-related in some small studies^39–41^, but was not reported as SUD-related in the GWAS catalog^36^.

### Brain dissection and cell type enrichment analyses.

For the 250 genes prioritized, we investigated in which brain dissections and cell types that they were significantly highly or lowly expressed. High-throughput single-cell RNA sequencing data from the Human Brain Cell Atlas v1.0 generated by the BRAIN Initiative cell census network^32^ were used. This data sampled 105 dissections from 10 brain regions across the forebrain, midbrain, and hindbrain, and identified 461 cell types. Prioritized genes were highly expressed in 35 dissections with 1 from amygdala, 27 from cortex, 2 from hippocampus, 3 from hypothalamus, and 2 from thalamus (**Supplemental Table 21**). Prioritized genes were lowly expressed in 2 dissections (one from pons and another from medulla, **Supplemental Table 22**). Prioritized genes were highly expressed in 125 of the 461 cell types, the majority of which are neuronal cells (**Supplemental Table 23**). Most of these cell types are from amygdala, basal forebrain, cerebral cortex, hippocampus, hypothalamus, and thalamus, in agreement with what were observed in the dissection enrichment analysis. Prioritized genes were lowly expressed in one cell type (splatter) from midbrain (**Supplemental Table 24**).

### Heritability estimation and heritability explained by SUD-concordant variants.

Due to the complicated LD patterns and mismatches of LD structures between the AA and LA samples and external LD reference panels, heritability estimation was performed only in EA samples. We identified 477,686 SUD-concordant variants in EA and using them, the estimated SNP heritability (*h*^2^_snp_) of SUDs (using LD score regression^42^) was 0.10 (SE = 0.001). SUD-concordant variants explained 46.6%, 53.0%, 59.1%, and 50.8% of *h*^2^_snp_ of PAU, OUD, CUD and NUD in EA (**Supplemental Table 25**).

### Genetic correlations.

We performed genetic correlation analyses using 1,151 GWAS summary statistics available at CTG-VL (https://vl.genoma.io/data). We identified 344 significant correlations after Bonferroni correction (**Supplemental Table 26**). Among them, 20 were related substance use, 44 were related to psychiatric diseases, 48 were related behavior, and 9 were related to life events. It is worth noting that while current alcohol drinker status was negatively correlated with SUDs (rG=−0.31, P-value = 3.09E-05), previous alcohol drinker status was positively correlated with SUDs (rG = 0.83, P-value = 1.64E-19).

### Polygenic scores (PGS) analyses.

We calculated PGS and tested their predictability in samples from the All of Us research program (Version 7.1) and the Indiana Biobank^33^. Since one of the major goals of PGS analyses is to identify high-risk individuals, we compared those top 5% individual with the highest PGS to everyone else. Additionally, since males and females have different prevalence of SUDs, we also performed sex-stratified analysis. In EA, the top 5% individuals were about two times more likely to have SUDs compared to everyone else in both All of Us (odds ratio (OR) = 2.21, 95% confidence interval (CI): 2.03–2.39) and the Indiana Biobank (OR = 1.88, 95%CI: 1.50–2.35), and PGS had larger ORs in females (All of US: OR = 2.35, 95%CI: 2.08–2.65; Indiana Biobank: OR = 2.03, 95%CI: 1.50–2.76) than in males (All of Us: OR = 2.07, 95%: 1.85–2.32; Indiana Biobank: OR = 1.71, 95%: 1.23–2.39) (Table 3). In LA, Indiana Biobank did not have sufficient sample size and thus PGS analyses were conducted in All of Us only. Those top 5% individuals were also approximately twice as likely to have SUDs (OR = 1.99, 95%CI: 1.71–2.33), but PGS had similar ORs in females (OR = 1.98, 95%CI: 1.65–2.51) and males (OR = 1.99, 95%CI: 1.62–2.44). In AA, PGS were not significant in any analysis (Table 3).

### Drug repurposing.

We queried the Drug Gene Interaction Database (DGIdb, v4.2.0)^43^ and identified 233 drugs approved by the Food and Drug Administration (FDA, targeting 29 genes; 278 drug-gene pairs). Among them, 93 drugs belong to the nerve system class of the Anatomical Therapeutic Chemical (ATC) Classification System (ATC code N, 159 drug-gene pairs, targeting 10 genes: *ANKK1, BDNF, CHRM4, DRD2, GABRA4, HTR3B, NCAM1, OPRD1, PDE4B,* and *POR*) (**Supplemental Table 27**). To identify potential repurposable drugs, we focused on drugs that: 1) have ATC code N, 2) are not approved to treat any SUD, 3) are shown to be related to SUDs treatment through literature search^44–49^, and 4) have comparators (i.e., drugs with the same ATC level 4 codes as drugs we identified but not targeting any gene we identified). Five drugs met these criteria: Topiramate in ATC N03AX (treating epilepsy, targeting *GABRA4*), Desipramine, Imipramine, and Nortriptyline in ATC N06AA (treating depression and anxiety, targeting *BDNF*) and Methylphenidate in ATC N06BA (treating ADHD, targeting *DRD2*). We used a large-scale real-world data from the Clinformatics^®^ (https://www.optum.com/business/life-sciences/commercial-analytics/managed-markets.html; sample sizes 325,542 to 1,817,258; **Supplemental Table 28**) to investigate whether these five drugs reduced the risk for SUDs. Compared to users of comparators, the hazard ratios for developing SUDs were: 0.44 for users of Topiramate (95%CI: 0.42–0.47), 0.89 (95% CI: 0.84–0.94) for users of Desipramine, Imipramine, or Nortriptyline; and 0.84 (95% CI: 0.78–0.91) for users of Methylphenidate, indicating that these five drugs significantly reduce the risk for SUDs and may be repurposed to treat SUDs.

## Discussion

In this study, using SUD-concordant and population-concordant variants, we identified 45 loci associated with SUDs in EA, AA, LA, and cross-ancestry meta-analyses. Through gene-based analyses, gene mapping, and gene prioritization, we identified 250 genes associated with SUDs. These genes are highly expressed in amygdala, cortex, hippocampus, hypothalamus, and thalamus, primarily in neuronal cells. SUD-concordant variants explained about 50% of heritability of each SUD in EA. The top 5% EA and LA individuals with the highest PGS calculated from SUD-concordant variants were about twice as likely to have SUDs compared to everyone else in their groups. PGS had larger odds ratios in females than in males in EA. We identified five FDA approved drugs to treat other diseases but could potentially be repurposed to treat SUDs.

Identifying SUD-shared genes is challenging. Each SUD is diagnosed by using multiple criteria and thus is heterogenous, and recent large-scale GWAS had demonstrated the highly polygenic nature of each SUD^10,12,22–27^. To search for SUD-shared gene, we combined all SUDs together as one phenotype thereby making it more heterogenous and more polygenic, consequently, an even larger sample size is needed. Furthermore, in large-scale SUD GWAS, some SUD-specific variants had extremely small P-values, and their P-values could remain genome-wide significant in multivariate analyses and often they were mistakenly identified as SUD-shared. Additionally, many variants irrelevant to SUDs (i.e., noises) could have small P-values (although not genome-wide significant) simply due to random variations. They usually were included in calculating PGS and thus decreased the PGS predictability. We conducted the largest multivariate analyses to date; and although we focused on concordant variants only, we identified more loci than previous studies^8,21^. Most importantly, by focusing on SUD-concordant variants, many SUD-specific variants and noises were excluded. This strategy was validated through the out-of-sample PGS analyses using two independent samples as the PGS had high predictability in EA and LA.

In our study, we replicated 14 of the 17 loci reported by Hatoum et al. in their multivariate analysis using the genomic SEM^8^. Of the 14 replicated loci, 11 had concordant effects and were genome-wide significant, three had concordant effects but were not genome-wide significant (rs2860846, EA P-value = 5.70E-06; rs55855024, EA P-value = 2.21E-06; and rs2861190, EA P-value = 1.88E-04). The three loci failed to replicate in our analyses had opposite directions of effects in some SUDs: rs1260326 (EA P-value = 2.07E-08, NUD had different direction), rs2424952 (EA P-value = 6.20E-04, CUD had different direction), and rs28567725 (EA P-value = 2.55E-07; AA P-value = 0.11; CUD and NUD had different directions of effects in both EA and AA). We also observed similar genetic correlations as those reported in Hatoum et al.,^8^ but we identified more significant genetic correlations and observed higher magnitude of estimated genetic correlations, likely due to the larger sample sizes used in this study. Overall, we confirmed most previous findings. The different results across studies are likely due to the different strategies used (meta-analyses and retaining concordant variants VS genomic SEM^8^), different phenotypes included (e.g., nicotine use disorder VS problematic tobacco use^8^), and larger sample size in our study.

Koob and Volkow proposed a heuristic framework of SUDs based on animal studies and human brain imaging data^3^. The framework involves three brain regions related to three stages of SUDs: basal ganglia (binge/intoxication stage), extended amygdala (withdrawal/negative affect stage), and prefrontal cortex (preoccupation/anticipation stage)^3^. We found that prioritized genes were highly expressed in prefrontal cortex, showing an overlap with the proposed framework. Furthermore, we found that among all 34 dissections from cortex, prioritized genes were highly expressed in 27 of them, warranting additional studies to characterize the role of cortex in SUDs. We did not find that the prioritized genes were highly expressed in any basal nuclei dissection after multiple testing correction, although caudate and putamen had unadjusted P-values 0.02 and 0.08, respectively. Prioritized genes were highly expressed in one amygdala dissection but not in extended amygdala. Note, while prioritized genes were not highly expressed in basal nuclei and extended amygdala, they were not lowly expressed in these two regions either. Therefore, they are not unrelated to SUDs but may play less important roles in SUDs than other regions. We found that prioritized genes were highly expressed mostly in neuronal cells, indicating that expressions of SUDs related genes are also cell type specific. Further studies are needed to confirm our findings.

In EA and LA, we found that PGS had odds ratios around 2, which is the suggested threshold for PGS to be incorporated in clinical settings to provide additional information for the risk evaluation^50^. For those at high-risk, early interventions could be applied to reduce the incident of SUDs, and personalized treatment could be developed to improve the probability of recovery. Additionally, PGS can be measured at any time, even before the initiation of risky substance use or risky behavior. All of these make it tempting to use PGS alone to identify high-risk individuals; however, we want to caution readers of problems of only using PGS. First, the estimated heritability of SUDs was ~ 50% and PGS only explained a portion of heritability; therefore, only using PGS will generate unreliable risk estimation. Second, unlike many diseases, the effect of PGS for SUDs could be significantly reduced or amplified by environmental factors across varying contexts. Some individuals having higher PGS may not use any substance due to their religions, cultures, and health conditions, or simply due to not having access to licit or illicit substances; therefore, PGS is meaningless for these individuals. On the other hand, some individuals with lower PGS may develop SUDs as a result of traumatic life events or psychiatric conditions and using PGS alone will overlook these individuals. To reliably predict the risk for SUDs, both PGS and environmental factors, as well as their interactions should be carefully considered.

We used a large-scale real-world data and a robust active comparator design to identify repurposable drugs. For those five drugs we identified, while previous studies have shown that they could be used to treat one or a few SUDs^44–49^; we showed that they could potentially be used to treat all SUDs as they are targeting SUD-shared genes. It is worth noting that four of the five drugs we identified (Desipramine, Imipramine, Nortriptyline, and Methylphenidate) target BDNF and DRD2–two genes are also the targets of drugs approved by FDA to treat SUDs such as Bupropion, Disulfiram, and Methadone. This may make them easier to get approval due to the well-understood mechanisms and these drugs should be prioritized for testing their efficacy in clinical trials.

There were limitations to this study. First, the sample sizes of both AA and LA discovery and target datasets are small, resulting in limited statistical power. Additionally in LA, we only have GWAS of AUD and OUD. Second, due to complicated LD patterns and different admixtures in AA and LA, we cannot accurately estimate heritability and genetic correlations in these populations. Third, we used fixed effect meta-analysis, which assumes the effect sizes are the same across different studies. This is a very strong assumption, especially for cross-ancestry analyses. Fourth, some variants may have different directions of effects for different SUDs and/or in different populations and they were filtered out by our strategy. Fifth, real-world data-based analysis were subject to unmeasured confounding and inconsistency between health insurance records and true health statues.

As addressed by de Hemptinne and Posthuma, genetic findings should not be taken out of context, misinterpreted, or misused^51^. In this study, we identified more variants in EA than in AA and LA, and the predictability of PGS were higher in EA than in AA and LA. We emphasis that these differences are due to much smaller sample sizes in AA and LA as well as the lack of appropriate statistical methods for admixed populations such as AA and LA. These differences do not suggest that AA and LA have different genetic mechanisms of SUDs. Rather, our results point to the critical and urgent need to increase the sample sizes of under-represented populations to reduce health disparities. We also emphasis that genetic factors or PGS (as an overall measure of effects of genetic factors) are not determination for SUD status. Genetic factors and environment factors such as traumatic life events and external stresses contribute about equally to SUDs. Findings from genetic studies must be interpreted with caution and cannot be used to predict SUD-related outcomes without carefully considering environment factors. Our findings should never be used to discriminate or to stigmatize people, or to deny access to prevention and treatment programs, especially for those from vulnerable populations. Our findings should only be used for research purposes to promote health.

In conclusion, using SUD-concordant and population-concordant variants, we have identified multiple genes associated with SUDs. These findings can help us elucidate the mechanisms of SUDs. PGS calculated using SUD-concordant variants had higher predictability in EA and LA. We also identified five drugs targeting genes associated with SUDs that may be repurposed to treat SUDs.

## Figures and Tables

**Table 1:** SUD GWAS included in meta-analyses.

Population	Trait	Diagnosis	# Samples	Authors	PMID
EA	Problematic alcohol use	DSM-IV, ICD9/10, AUDIT-P	435,563	Zhou et al 2020	32451486
	Opioid use disorder	DSM-IV, ICD9/10	308,733	Polimanti et al 2020, Kember et al 2022	32099098, 36171425
	Cannabis use disorder	DSM-IV, ICD9/10	374,287	Johnson et al 2020	33096046
	Nicotine use disorder	ICD10, FTND	291,103	Watanabe et al 2019, Quach et al 2020	31427789, 33144568
	FinnGen Substance abuse	ICD9/10	251,534	NA	NA
	Total		1,467,929		
AA	Alcohol use disorder	DSM-IV, ICD9/10	62,928	Walters et al 2018, Kranzler et al 2019	30482948, 30940813
	Opioid use disorder	DSM-IV, ICD9/10	91,026	Polimanti et al 2020, Kember et al 2022	32099098, 36171425
	Cannabis use disorder	DSM-IV, ICD9/10	9,745	Johnson et al 2020	33096046
	Nicotine use disorder	FTND	11,787	Quach et al 2020	33144568
	Total		159,000		
LA	Alcohol use disorder	ICD9/10	14,175	Kranzler et al 2019	30940813
	Opioid use disorder	ICD9/10	34,861	Kember et al 2020	36171425
	Total		45,757		
Cross ancestry total			1,672,656		

Note: the total samples sizes are effective sample sizes, i.e., samples sizes after correcting sample overlapping. PMID: PubMed ID; AUDIT-P: problematic subscale of the Alcohol Use Disorder Identification Test; FTND: Fagerstrom Test for Nicotine Dependence. AA: African ancestry. EA: European ancestry. LA: Latino ancestry.

**Table 2:** Genome-wide significant loci and their lead variants.

Locus	Lead variant	Chr	BP	Effect Allele	Non-Effect Allele	Z	P value	Population	Locus start	Locus end	Additional population
1	rs4233254	1	29,168,054	T	C	5.627	1.83E-08	EA	28,634,681	29,169,593	EA_LA
2	rs6690101	1	66,414,039	T	C	-7.891	3.00E-15	EA	66,334,518	66,547,212	AA EA, EA_LA, AA_EA_LA
3	rs2340403	1	73,835,777	T	C	−6.775	1.24E-11	AA_EA	73,766,431	74,002,104	EA, EA LA, AA_EA_LA
4	rs66994942	2	22,577,783	A	G	6.55	5.76E-11	EA	22,430,795	22,971,097	AA_EA
5	rs472140	2	45,139,904	T	C	6.591	4.36E-11	EA_LA	45,121,466	45,175,585	EA, AA_EA
6	rs11309539	2	58,025,739	T	TA	5.576	2.46E-08	AA_EA	57,942,987	58,444,610	EA
7	rs10865306	2	58,913,139	T	C	−5.51	3.59E-08	EA	58,863,670	58,932,674	
8	rs4625930	2	101,249,256	A	G	−5.813	6.14E-09	EA	101,232,647	101,317,319	EA_LA
9	rs35942385	2	144,208,523	T	G	−5.792	6.94E-09	EA_LA	144,145,478	144,263,280	EA
10	rs9812360	3	49,402,351	T	C	6.601	4.09E-11	AA_EA	48,724,599	49,890,967	EA, EA LA, AA_EA_LA
11	rs1694933	3	84,951,716	A	C	−5.996	2.02E-09	AA_EA	84,853,161	84,951,716	EA
12	rs62250713	3	85,513,793	A	G	5.75	8.95E-09	EA	85,403,982	85,671,909	EA_LA
13	rs6824152	4	46,915,161	T	C	−5.618	1.93E-08	AA_EA_LA	46,879,127	46,972,899	AA_EA
14	rs1229984	4	100,239,319	T	C	−5.512	3.55E-08	LA	100,239,319	100,239,319	
15	rs13109404	4	102,896,591	T	G	6	1.97E-09	EA_LA	102,702,364	103,001,649	EA
16	rs72678864	4	112,422,145	A	G	−5.841	5.19E-09	EA	112,303,764	112,503,872	EA_LA
17	rs4413540	5	30,814,680	T	C	5.468	4.54E-08	EA	30,813,302	30,842,054	
18	rs71627581	5	43,161,351	A	G	−6.69	2.23E-11	AA_EA	43,125,795	43,190,647	EA
19	rs40506	5	60,525,210	T	C	5.592	2.24E-08	EA	60,484,179	60,614,879	
20	rs61258990	6	19,278,344	CT	C	−5.668	1.45E-08	EA	19,211,776	19,358,341	
21	rs73377013	6	22,200,119	T	C	5.771	7.87E-09	LA	21,318,867	22,702,294	
22	rs115133207	6	24,394,925	T	C	5.492	3.98E-08	LA	24,355,002	24,427,763	
23	rs1796520	6	26,410,800	T	C	−6.529	6.63E-11	EA_LA	26,364,111	26,577,186	EA
24	rs4713121	6	27,722,064	T	C	5.578	2.44E-08	EA_LA	27,347,808	27,980,220	EA, AA_EA
25	rs6467958	7	75,622,281	T	C	−6.242	4.33E-10	EA	75,607,155	75,852,480	AA EA, EA_LA, AA_EA_LA
26	rs2189010	7	114,119,430	A	G	7.433	1.06E-13	EA	113,981,217	114,290,415	AA EA, EA_LA, AA_EA_LA
27	rs10264648	7	114,956,168	T	C	5.496	3.88E-08	EA	114,940,159	115,025,708	
28	rs2551777	7	135,100,476	T	C	−6.075	1.24E-09	EA_LA	135,050,259	135,221,170	EA
29	rs72671424	8	93,056,264	A	G	6.501	7.97E-11	EA	92,976,563	93,180,965	
30	rs12345339	9	12,369,424	T	C	−5.642	1.69E-08	EA	12,358,209	12,432,665	
31	rs405039	9	17,076,950	A	C	5.778	7.55E-09	EA_LA	17,044,774	17,127,924	EA
32	rs12380310	9	127,973,473	A	G	−6.533	6.47E-11	EA	127,780,246	128,008,537	EA_LA
33	rs7044246	9	134,866,271	T	C	−5.509	3.61E-08	EA	134,802,170	134,946,805	
34	rs1591660	10	110,500,212	T	C	−5.514	3.50E-08	EA_LA	110,462,847	110,635,222	
35	rs7476	11	46,342,834	A	C	−5.641	1.69E-08	EA	46,336,995	46,705,193	
36	rs11229119	11	57,535,966	T	C	−6.179	6.44E-10	EA	57,409,538	57,756,568	
37	rs1116313	11	113,296,107	A	G	8.473	2.39E-17	AA_EA_LA	112,826,867	113,692,660	EA, AA_EA,EA_LA
38	rs647905	11	121,534,938	T	C	6.215	5.14E-10	EA_LA	121,495,141	121,553,314	EA, AA_EA,AA_EA_LA
39	rs11432080	13	96,970,919	G	GT	−5.645	1.65E-08	EA	96,901,225	97,029,792	
40	rs6576007	14	104,323,777	T	G	−8.329	8.18E-17	EA	103,991,478	104,363,528	AA EA, EA_LA, AA_EA_LA
41	rs12908163	15	47,641,522	T	C	7.726	1.11E-14	EA	47,613,403	47,685,416	AA EA, EA_LA, AA_EA_LA
42	rs12232639	18	26,570,703	A	G	−5.525	3.29E-08	EA	26,570,584	26,611,564	AA_EA,AA_EA_LA
43	rs2613765	19	5,066,330	A	G	−5.705	1.17E-08	AA_EA	5,046,070	5,086,979	EA
44	rs62128800	19	10,667,750	T	C	5.776	7.64E-09	AA_EA	10,648,933	10,686,769	
45	rs1231281	19	49,239,200	A	G	6.098	1.08E-09	EA_LA	49,206,108	49,259,529	EA

Note: Chr: chromosome; BP: base pair positions. AA: African ancestry. EA: European ancestry. LA: Latino ancestry.

**Table 3:** PGS analyses results.

Sample	Population	Sex	TotalN	Total N.case(%)	Total N.control(%)	Top5% N	Top5% N.case(%)	Top5% N.control(%)	Estimate	StdError	OR (95%CI)	P-value
AOU	AA	Both	47,516	6,090 (12.82%)	41,426 (87.18%)	2,303	292 (12.68%)	2,011 (87.32%)	−0.01	0.07	0.99 (0.87–1.13)	0.92
AOU	AA	Male	20,989	3,699 (17.62%)	17,290 (82.38%)	1,009	181 (17.94%)	828 (82.06%)	0.00	0.09	1.00 (0.84–1.19)	0.98
AOU	AA	Female	26,527	2,391 (9.01%)	24,136 (90.99%)	1,294	111 (8.58%)	1,183 (91.42%)	−0.08	0.11	0.93 (0.75–1.14)	0.48
**AOU**	**EA**	**Both**	**116,027**	**7,672 (6.61%)**	**108,355 (93.39%)**	**5,710**	**792 (13.87%)**	**4,918 (86.13%)**	**0.79**	**0.04**	**2.21 (2.03–2.39)**	**5.5580**
**AOU**	**EA**	**Male**	**45,919**	**4,405 (9.59%)**	**41,514 (90.41%)**	**2,365**	**451 (19.07%)**	**1,914 (80.93%)**	**0.73**	**0.06**	**2.07 (1.85–2.32)**	**5.3937**
**AOU**	**EA**	**Female**	**70,108**	**3,267 (4.66%)**	**66,841 (95.34%)**	**3,345**	**341 (10.19%)**	**3,004 (89.81%)**	**0.86**	**0.06**	**2.35 (2.08–2.65)**	**1.0443**
**AOU**	**LA**	**Both**	**36,368**	**2,461 (6.77%)**	**33,907 (93.23%)**	**1,818**	**217 (11.94%)**	**1,601 (88.06%)**	**0.69**	**0.08**	**1.99 (1.71–2.33)**	**3.2018**
**AOU**	**LA**	**Male**	**12,358**	**1,543 (12.49%)**	**10,815 (87.51%)**	**646**	**134 (20.74%)**	**512 (79.26%)**	**0.69**	**0.1**	**1.99 (1.62–2.44)**	**3.5411**
**AOU**	**LA**	**Female**	**24,010**	**918 (3.82%)**	**23,092 (96.18%)**	**1,172**	**83 (7.08%)**	**1,089 (92.92%)**	**0.68**	**0.12**	**1.98 (1.56–2.51)**	**2.2008**
IB	AA	Both	1,829	912 (49.86%)	917 (50.14%)	158	77 (48.73%)	81 (51.27%)	−0.01	0.2	0.99 (0.66–1.47)	0.96
IB	AA	Male	647	371 (57.34%)	276 (42.66%)	54	28 (51.85%)	26 (48.15%)	−0.02	0.36	0.98 (0.48–1.98)	0.95
IB	AA	Female	1,182	541 (45.77%)	641 (54.23%)	104	49 (47.12%)	55 (52.88%)	−0.02	0.25	0.98 (0.60–1.61)	0.95
**IB**	**EA**	**Both**	**6,375**	**2,801 (43.94%)**	**3,574 (56.06%)**	**406**	**248 (61.08%)**	**158 (38.92%)**	**0.63**	**0.11**	**1.88 (1.50–2.35)**	**3.3708**
**IB**	**EA**	**Male**	**2,862**	**1,301 (45.46%)**	**1,561 (54.54%)**	**180**	**110 (61.11%)**	**70 (38.89%)**	**0.54**	**0.17**	**1.71 (1.23–2.39)**	**1.5603**
**IB**	**EA**	**Female**	**3,513**	**1,500 (42.70%)**	**2,013 (57.30%)**	**226**	**138 (61.06%)**	**88 (38.94%)**	**0.71**	**0.15**	**2.03 (1.50–2.76)**	**4.4106**

Note: N.case: number of cases; N.control: number of controls. Significant P-values are in bold. AOU: All of Us. IB: Indiana Biobank. AA: African ancestry. EA: European ancestry. LA: Latino ancestry.

## Data Availability

EA problematic alcohol use GWAS (Zhou et al 2020), EA, AA, and LA opioid use disorder GWAS (Kember et al 2022), AA alcohol use disorder (Kranzler et al 2019) are available through dbGaP: https://www.ncbi.nlm.nih.gov/projects/gap/cgi-bin/study.cgi?study_id=phs001672. EA and AA opioid use disorder GWAS (Polimanti et al 2020): https://figshare.com/articles/dataset/sud2020-op/14672211. EA and AA cannabis use disorder GWAS (Johnson et al 2020): https://figshare.com/articles/dataset/sud2020-cud/14842692. EA nicotine use disorder GWAS (Watanabe et al 2019): https://atlas.ctglab.nl/ukb2_sumstats/41204_F17_logistic.EUR.sumstats.MACfilt.txt.gz. EA and AA nicotine use disorder GWAS (Quach et al 2022) are available through dbGaP: https://www.ncbi.nlm.nih.gov/projects/gap/cgi-bin/study.cgi?study_id=phs001532.v2.p1. FinnGen Substance abuse: https://www.finngen.fi/en/access_results. AA alcohol use disorder (Walters et al 2018): https://figshare.com/articles/dataset/sud2018-alc/14672187. The Human Brain Cell Atlas v1.0 generated by BRAIN Initiative cell census network: https://cellxgene.cziscience.com/collections/283d65eb-dd53-496d-adb7-7570c7caa443. All of Us research program: https://www.researchallofus.org/data-tools/workbench/. Indiana Biobank: https://indianabiobank.org/. Indiana biobank substance use disorder cohort is also available from dbGaP: https://www.ncbi.nlm.nih.gov/projects/gap/cgi-bin/study.cgi?study_id=phs003025.v1.p1. All GWAS summary statistics from this study will be deposited to GWAS catalog: https://www.ebi.ac.uk/gwas/. All variants and their weights used to calculated PGS will be available from PGS catalog: https://www.pgscatalog.org/.

## References

[1] Degenhardt L., The global burden of disease attributable to alcohol and drug use in 195 countries and territories, 1990–2016: a systematic analysis for the Global Burden of Disease Study 2016. The Lancet Psychiatry 5, 987–1012 (2018). 30392731 10.1016/S2215-0366(18)30337-7 PMC6251968

[2] Deak J.D. & Johnson E.C. Genetics of substance use disorders: a review. Psychol Med 51, 2189–2200 (2021). 33879270 10.1017/S0033291721000969 PMC8477224

[3] Koob G.F. & Volkow N.D. Neurobiology of addiction: a neurocircuitry analysis. Lancet Psychiatry 3, 760–773 (2016). 27475769 10.1016/S2215-0366(16)00104-8 PMC6135092

[4] Bhalla I.P., Stefanovics E.A. & Rosenheck R.A. Clinical Epidemiology of Single Versus Multiple Substance Use Disorders: Polysubstance Use Disorder. Medical Care 55(2017). 10.1097/MLR.0000000000000731 28806363

[5] Kendler K.S., Jacobson K.C., Prescott C.A. & Neale M.C. Specificity of Genetic and Environmental Risk Factors for Use and Abuse/Dependence of Cannabis, Cocaine, Hallucinogens, Sedatives, Stimulants, and Opiates in Male Twins. American Journal of Psychiatry 160, 687–695 (2003). 12668357 10.1176/appi.ajp.160.4.687

[6] Kendler K.S., Myers J. & Prescott C.A. Specificity of Genetic and Environmental Risk Factors for Symptoms of Cannabis, Cocaine, Alcohol, Caffeine, and Nicotine Dependence. Arch Gen Psychiat 64, 1313–1320 (2007). 17984400 10.1001/archpsyc.64.11.1313

[7] Kendler K.S., Recent advances in the genetic epidemiology and molecular genetics of substance use disorders. Nature Neuroscience 15, 181–189 (2012). 22281715 10.1038/nn.3018 PMC3297622

[8] Hatoum A.S., Multivariate genome-wide association meta-analysis of over 1 million subjects identifies loci underlying multiple substance use disorders. Nature Mental Health 1, 210–223 (2023). 37250466 10.1038/s44220-023-00034-y PMC10217792

[9] Hatoum A.S., The addiction risk factor: A unitary genetic vulnerability characterizes substance use disorders and their associations with common correlates. Neuropsychopharmacology 47, 1739–1745 (2022). 34750568 10.1038/s41386-021-01209-w PMC9372072

[10] Zhou H., Genome-wide meta-analysis of problematic alcohol use in 435,563 individuals yields insights into biology and relationships with other traits. Nature Neuroscience 23, 809–818 (2020). 32451486 10.1038/s41593-020-0643-5 PMC7485556

[11] Xu H., Identifying genetic loci and phenomic associations of substance use traits: A multi-trait analysis of GWAS (MTAG) study. Addiction (2023). 10.1111/add.16229 PMC10754226 37156939

[12] Kranzler H.R., Genome-wide association study of alcohol consumption and use disorder in 274,424 individuals from multiple populations. Nature Communications 10, 1499 (2019). 10.1038/s41467-019-09480-8 PMC6445072 30940813

[13] Deak J.D., Genome-wide association study in individuals of European and African ancestry and multi-trait analysis of opioid use disorder identifies 19 independent genome-wide significant risk loci. Mol Psychiatr (2022). 10.1038/s41380-022-01709-1 PMC9718667 35879402

[14] Gaddis N., Multi-trait genome-wide association study of opioid addiction: OPRM1 and beyond. Sci Rep 12, 16873 (2022). 36207451 10.1038/s41598-022-21003-y PMC9546890

[15] Johnson E.O., KAT2B polymorphism identified for drug abuse in African Americans with regulatory links to drug abuse pathways in human prefrontal cortex. Addict Biol 21, 1217–1232 (2016). 26202629 10.1111/adb.12286 PMC4724343

[16] Xiang B., Yang B.Z., Zhou H., Kranzler H. & Gelernter J. GWAS and network analysis of co-occurring nicotine and alcohol dependence identifies significantly associated alleles and network. Am J Med Genet B Neuropsychiatr Genet 180, 3–11 (2019). 30488612 10.1002/ajmg.b.32692 PMC6918694

[17] Wetherill L., Genome-wide association study identifies loci associated with liability to alcohol and drug dependence that is associated with variability in reward-related ventral striatum activity in African- and European-Americans. Genes Brain Behav 18, e12580 (2019). 31099175 10.1111/gbb.12580 PMC6726116

[18] Chang S.H., Genome-wide association meta-analyses identify novel genetic risk loci and polygenic phenotype associations for heroin, methamphetamine and alcohol dependences. Clin Transl Med 12, e659 (2022). 35075802 10.1002/ctm2.659 PMC8787099

[19] Zhang H., Strong and weak cross-inheritance of substance use disorders in a nationally representative sample. Mol Psychiatr 27, 1742–1753 (2022). 10.1038/s41380-021-01370-0 PMC9085976 34759357

[20] Grotzinger A.D., Genomic structural equation modelling provides insights into the multivariate genetic architecture of complex traits. Nature Human Behaviour 3, 513–525 (2019). 10.1038/s41562-019-0566-x PMC6520146 30962613

[21] Schoeler T., Novel Biological Insights Into the Common Heritable Liability to Substance Involvement: A Multivariate Genome-wide Association Study. Biol Psychiat 93, 524–535 (2023).

[22] Walters R.K., Transancestral GWAS of alcohol dependence reveals common genetic underpinnings with psychiatric disorders. Nat Neurosci 21, 1656–1669 (2018). 30482948 10.1038/s41593-018-0275-1 PMC6430207

[23] Johnson E.C., A large-scale genome-wide association study meta-analysis of cannabis use disorder. Lancet Psychiatry 7, 1032–1045 (2020). 33096046 10.1016/S2215-0366(20)30339-4 PMC7674631

[24] Kember R.L., Cross-ancestry meta-analysis of opioid use disorder uncovers novel loci with predominant effects in brain regions associated with addiction. Nature Neuroscience 25, 1279–1287 (2022). 36171425 10.1038/s41593-022-01160-z PMC9682545

[25] Polimanti R., Leveraging genome-wide data to investigate differences between opioid use vs. opioid dependence in 41,176 individuals from the Psychiatric Genomics Consortium. Mol Psychiatry 25, 1673–1687 (2020). 32099098 10.1038/s41380-020-0677-9 PMC7392789

[26] Watanabe K., A global overview of pleiotropy and genetic architecture in complex traits. Nat Genet 51, 1339–1348 (2019). 31427789 10.1038/s41588-019-0481-0

[27] Quach B.C., Expanding the genetic architecture of nicotine dependence and its shared genetics with multiple traits. Nat Commun 11, 5562 (2020). 33144568 10.1038/s41467-020-19265-z PMC7642344

[28] Solovieff N., Cotsapas C., Lee P.H., Purcell S.M. & Smoller J.W. Pleiotropy in complex traits: challenges and strategies. Nature Reviews Genetics 14, 483–495 (2013). 10.1038/nrg3461 PMC4104202 23752797

[29] Zhu Z., Anttila V., Smoller J.W. & Lee P.H. Statistical power and utility of meta-analysis methods for cross-phenotype genome-wide association studies. PLOS ONE 13, e0193256 (2018). 29494641 10.1371/journal.pone.0193256 PMC5832233

[30] Lee P.H., Genomic Relationships, Novel Loci, and Pleiotropic Mechanisms across Eight Psychiatric Disorders. Cell 179, 1469–1482.e1411 (2019). 31835028 10.1016/j.cell.2019.11.020 PMC7077032

[31] Lonsdale J., The Genotype-Tissue Expression (GTEx) project. Nature Genetics 45, 580–585 (2013). 23715323 10.1038/ng.2653 PMC4010069

[32] Siletti K., Transcriptomic diversity of cell types across the adult human brain. Science 382, eadd7046 (2023). 37824663 10.1126/science.add7046

[33] Lai D., Gene-based polygenic risk scores analysis of alcohol use disorder in African Americans. Transl Psychiatry 12, 266 (2022). 35790736 10.1038/s41398-022-02029-2 PMC9256707

[34] Kurki M.I., FinnGen provides genetic insights from a well-phenotyped isolated population. Nature 613, 508–518 (2023). 36653562 10.1038/s41586-022-05473-8 PMC9849126

[35] Edenberg H.J. & McClintick J.N. Alcohol Dehydrogenases, Aldehyde Dehydrogenases, and Alcohol Use Disorders: A Critical Review. Alcoholism-Clinical and Experimental Research 42, 2281–2297 (2018). 30320893 10.1111/acer.13904 PMC6286250

[36] Sollis E., The NHGRI-EBI GWAS Catalog: knowledgebase and deposition resource. Nucleic Acids Res 51, D977–d985 (2023). 36350656 10.1093/nar/gkac1010 PMC9825413

[37] de Leeuw C.A., Mooij J.M., Heskes T. & Posthuma D. MAGMA: generalized gene-set analysis of GWAS data. PLoS Comput Biol 11, e1004219 (2015). 25885710 10.1371/journal.pcbi.1004219 PMC4401657

[38] Szklarczyk D., The STRING database in 2021: customizable protein-protein networks, and functional characterization of user-uploaded gene/measurement sets. Nucleic Acids Res 49, D605–d612 (2021). 33237311 10.1093/nar/gkaa1074 PMC7779004

[39] Burns J.A., Molecular Imaging of Opioid and Dopamine Systems: Insights Into the Pharmacogenetics of Opioid Use Disorders. Front Psychiatry 10, 626 (2019). 31620026 10.3389/fpsyt.2019.00626 PMC6759955

[40] Nelson E.C., Association of OPRD1 polymorphisms with heroin dependence in a large case-control series. Addict Biol 19, 111–121 (2014). 22500942 10.1111/j.1369-1600.2012.00445.x PMC3867542

[41] Levran O., Randesi M., Adelson M. & Kreek M.J. OPRD1 SNPs associated with opioid addiction are cis-eQTLs for the phosphatase and actin regulator 4 gene, PHACTR4, a mediator of cytoskeletal dynamics. Transl Psychiatry 11, 316 (2021). 34031368 10.1038/s41398-021-01439-y PMC8144180

[42] Finucane H.K., Partitioning heritability by functional annotation using genome-wide association summary statistics. Nature Genetics 47, 1228-+ (2015). 26414678 10.1038/ng.3404 PMC4626285

[43] Freshour S.L., Integration of the Drug-Gene Interaction Database (DGIdb 4.0) with open crowdsource efforts. Nucleic Acids Res 49, D1144–D1151 (2021). 33237278 10.1093/nar/gkaa1084 PMC7778926

[44] Kranzler H.R., Topiramate treatment for heavy drinkers: moderation by a GRIK1 polymorphism. Am J Psychiatry 171, 445–452 (2014). 24525690 10.1176/appi.ajp.2013.13081014 PMC3997125

[45] Johnson B.A., Topiramate for the treatment of cocaine addiction: a randomized clinical trial. JAMA Psychiatry 70, 1338–1346 (2013). 24132249 10.1001/jamapsychiatry.2013.2295

[46] Kosten T., Desipramine and contingency management for cocaine and opiate dependence in buprenorphine maintained patients. Drug Alcohol Depend 70, 315–325 (2003). 12757969 10.1016/s0376-8716(03)00032-2

[47] Agabio R., Trogu E. & Pani P.P. Antidepressants for the treatment of people with co-occurring depression and alcohol dependence. Cochrane Database Syst Rev 4, Cd008581 (2018). 29688573 10.1002/14651858.CD008581.pub2 PMC6494437

[48] Prochazka A.V., A randomized trial of nortriptyline for smoking cessation. Arch Intern Med 158, 2035–2039 (1998). 9778204 10.1001/archinte.158.18.2035

[49] Aryan N., The therapeutic effects of methylphenidate and matrix-methylphenidate on addiction severity, craving, relapse and mental health in the methamphetamine use disorder. Subst Abuse Treat Prev Policy 15, 72 (2020). 32977820 10.1186/s13011-020-00317-y PMC7519552

[50] Hao L., Development of a clinical polygenic risk score assay and reporting workflow. Nat Med (2022). 10.1038/s41591-022-01767-6 PMC9117136 35437332

[51] de Hemptinne M.C. & Posthuma D. Addressing the ethical and societal challenges posed by genome-wide association studies of behavioral and brain-related traits. Nature Neuroscience 26, 932–941 (2023). 37217727 10.1038/s41593-023-01333-4

